# Supplementary material for: The interactome of intact mitochondria by cross-linking mass spectrometry provides evidence for coexisting respiratory supercomplexes
Source: Mol Cell Proteomics. 2017 Dec 8;17(2):216–32. doi: 10.1074/mcp.RA117.000470 (PMC5795388; doi:10.1074/mcp.RA117.000470)
Supplement: Supplemental Data [file supp_RA117.000470_133922_0_supp_23978_fzffwf.zip › spectra_annotation/mito_DR_spectra_annotation/313-1-7-1-8-1.pdf]

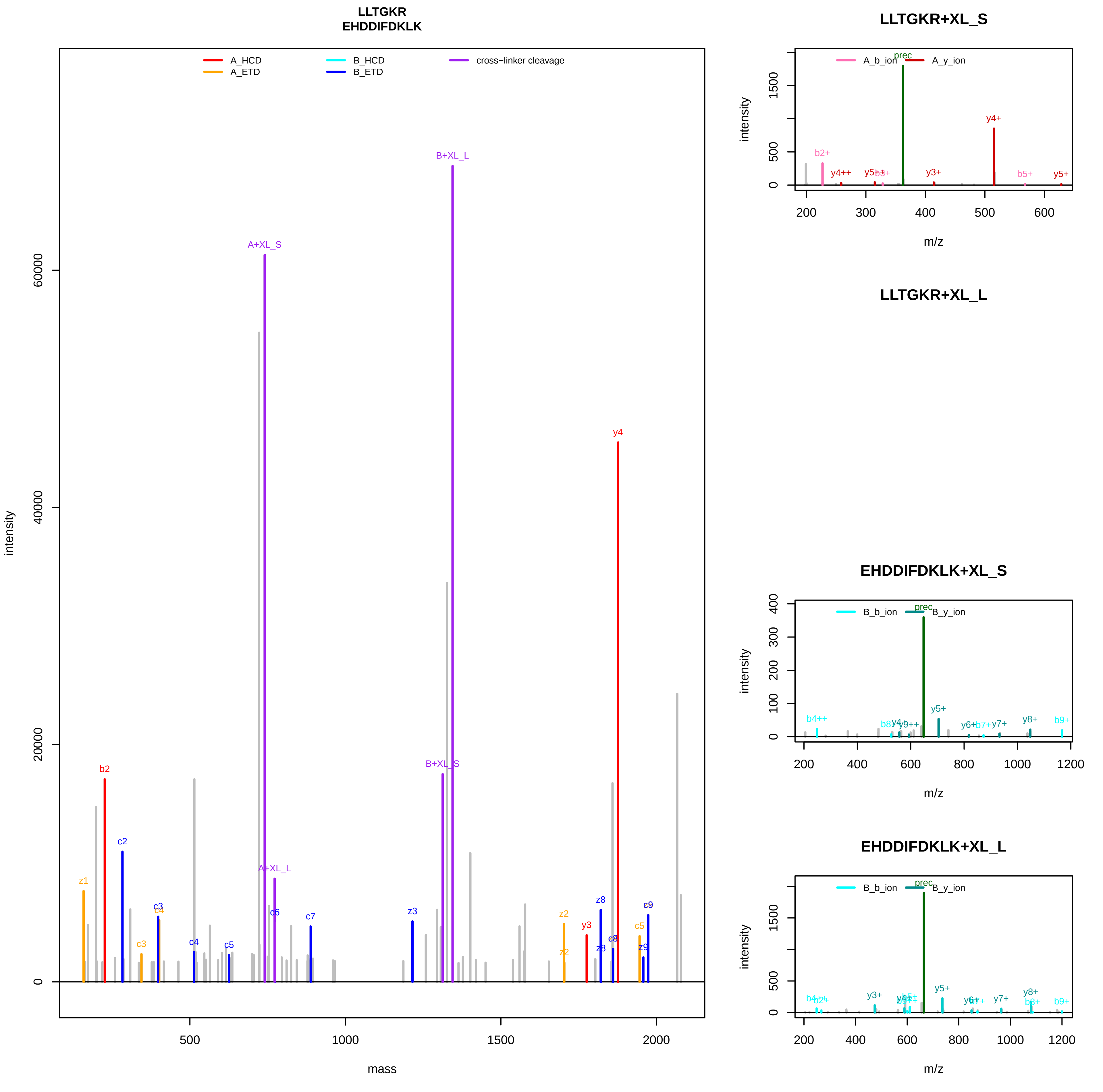

LLTGKR
EHDDIFDKLK
A_HCD
A_ETD
B_HCD
B_ETD
cross−linker cleavage
B+XL_L
A+XL_S
y4
b2
B+XL_S
c2
A+XL_L
z1
c3
c4
c6
c7
z3
z8
c9
z2
y3
c5
c8
z9
c5
c3
c4
intensity
mass
LLTGKR+XL_S
A_b_ion
A_y_ion
prec
b2+
y4++
y5++
b3+
y3+
y4+
b5+
y5+
m/z
LLTGKR+XL_L
EHDDIFDKLK+XL_S
B_b_ion
B_y_ion
b4++
y5+
y6+
b7+
y7+
y8+
b9+
EHDDIFDKLK+XL_L
y3+
